# Morphological Characterization of Intrafollicular Epithelial Bodies (IFEBs) in Rabbit Peyer’s Patches

**DOI:** 10.3390/ijms26073207

**Published:** 2025-03-30

**Authors:** Tiziana Tamborrino, Denise Bonente, Marì Regoli, Valentina Costa, Virginia Barone, Emiliana Giacomello, Giulia Collodel, Niccolò Fagni, Claudio Nicoletti, Eugenio Bertelli

**Affiliations:** 1 Department of Molecular and Developmental Medicine, University of Siena, Via A. Moro, 2, 53100 Siena, Italy; tiziana.tamborrino@student.unisi.it (T.T.); denise.bonente@student.unisi.it (D.B.); v.costa@student.unisi.it (V.C.); virginia.barone@unisi.it (V.B.); giulia.collodel@unisi.it (G.C.); 2 Department of Life Sciences, University of Siena, Via A. Moro, 2, 53100 Siena, Italy; 3 Department of Medicine, Surgery and Health Sciences, University of Trieste, Strada Fiume, 447, 34149 Trieste, Italy; egiacomello@units.it; 4 Otorinolaringoiatry, AOUS (Azienda Ospedaliero-Universitaria Senese), 53100 Siena, Italy; niccofagni93@gmail.com; 5 Department of Medicine, University of Udine, Via Colugna 50, 33100 Udine, Italy

**Keywords:** follicle-associated epithelium, M cells, intrafollicular epithelial body, entactin, basement membrane, anoikis, cell polarity

## Abstract

Follicle-associated epithelium (FAE) covering the lymphoid follicles of Peyer’s patches (PPs) plays a central role in mucosal immunity. Here, we investigated FAE-derived intrafollicular epithelial bodies (IFEBs) that apparently detach from the FAE and sink deep into the lymphoid tissue of the PPs. Analysis of rabbit PP FAE was carried out by a variety of microscopy and immunohistochemistry techniques using a panel of specific antibodies to determine the nature of the IFEBs. IFEBs displayed the typical features of the FAE, with cytokeratin (CK)+ epithelial cells and CK+/vimentin+ M-cell-like cells. Serial sections of PP tissues showed that the IFEBs are formations frequently separated by the overlying FAE that maintains its integrity. Further, IFEBs showed the presence of junction-associated molecules like zonulin-1 and desmoplakins. Also, IFEBs apparently disaggregate within the lymphoid tissue, as demonstrated by basement membrane disappearance and the finding of isolated epithelial cells that acquire the features of non-polarized epithelial cells. Segments of the FAE in rabbit PPs can detach, forming IFEBs that migrate inside the lymphoid tissue. Although the biological relevance of the newly described IFEBs remains to be determined, we interpreted these data as showing the highly dynamic nature of the PP-associated FAE.

## 1. Introduction

Peyer’s patches (PPs) are lymphoid follicle aggregates present along the mammalian gut. PPs, along with solitary lymphoid follicles and the appendix, are referred to as gut-associated lymphoid tissue, which is one major component of the mucosal immune system. Every lymphoid follicle, either solitary or belonging to PPs and appendix, displays the same structure, characterized by specific lymphoid cell populations restricted to particular areas of the follicle underlying a specialized follicle-associated epithelium (FAE) [1]. The latter differs from the conventional villus epithelium both morphologically and functionally. Indeed, in addition to conventional absorptive enterocytes, the FAE is formed by a limited number of goblet and entero-endocrine cells and, most notably, specialized membranous (M) cells [2]. Although also present in some villi [3], M cells are mainly located within the FAE, and their number considerably varies among mammalian species [4]. The FAE also differs from the normal intestinal epithelium by its ability to accommodate a large and diverse population of immune cells [5]. These cells are harbored inside M-cell hollows, just beneath the cell apex, that in some animals, like the rabbit, create an intricate network of an expanded space, which could theoretically extend throughout the entire FAE. Thanks to M cells that are able to uptake various materials from the gut lumen and to convey them to the underlying dome area, the FAE functions preferentially as a “sampler” epithelium. This M-cell ability has been widely studied, and these cells have been reported to be able to uptake and internalize inorganic materials, viruses, bacteria and soluble molecules, including prions [6,7].

In the past years, several studies demonstrated the highly dynamic nature of the FAE that undergoes a rapid remodeling upon antigenic stimulation [8,9,10,11,12]. Taken together, these studies demonstrated that the FAE population can rapidly change in the presence of specific antigenic stimuli. These rapid changes included an increased number of M-cell-infiltrating immune cells [8,9,11,12], antigen-dependent increase in M-cell size [8] and increase in M-cell number following challenge with *S. typhimurium* aro A- and *S. pneumoniae* [9,11,12,13]. Further, studies identified a series of molecules underpinning these events, including macrophage inhibitory factor (MIF) and the ligand of the receptor activator of nuclear factor κB (RANKL) [14,15]. Thus, the FAE is a very plastic epithelium, capable of responding to either exogenous (i.e., bacteria) or endogenous stimuli, such as soluble molecules from the underlying immune microenvironment.

Here, we report for the first time that, in rabbit PPs, a high percentage of lymphoid follicles of naïve rabbits host isolated intrafollicular epithelial bodies (IFEBs) directly originated from the FAE. These fragments of the FAE, at times very large, were found embedded within the dome area of the lymphoid follicles and, in most cases, have lost their continuity with the overlying FAE. IFEBs display diverse morphological aspects, ranging from micropseudocyst- to labyrinth-like, and are often associated with nearby isolated epithelial cells. They are formed by viable, non-apoptotic cells that appear to change their morphology as they relocate within the follicles. Although the function of these newly described structures remains to be determined, the presence of IFEB within the lymphoid tissue of PPs suggests a potential role in the intestinal immune regulation.

## 2. Results

**Figure 1:**
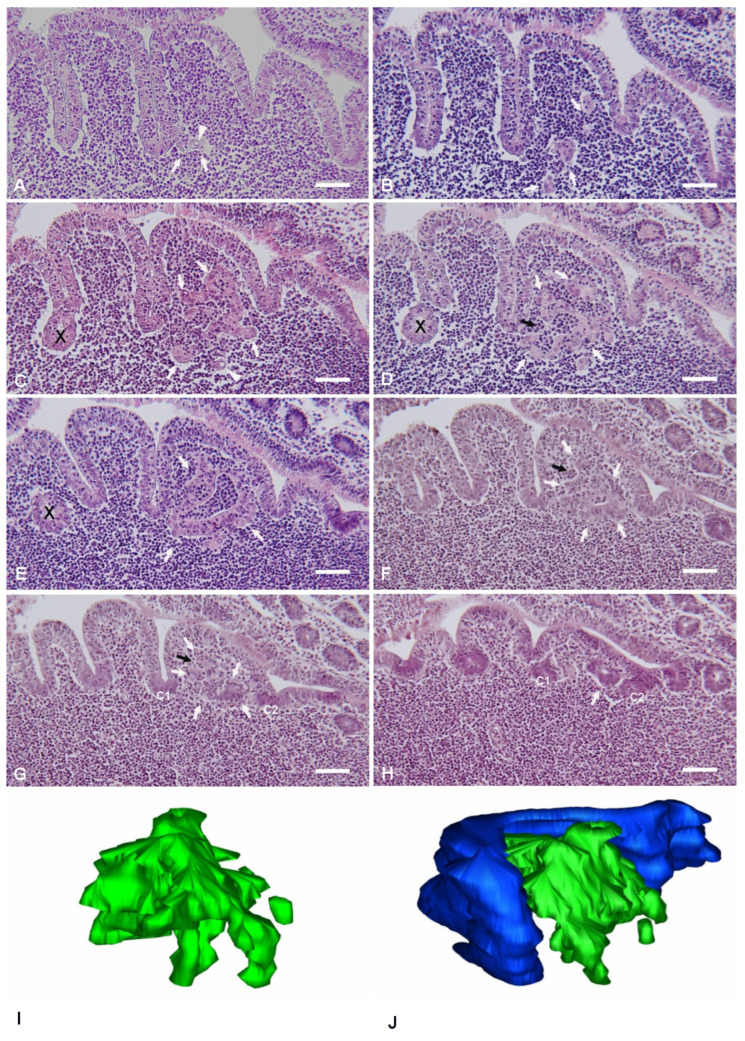
IFEBs. Selected slides from a series of consecutive sections (**A**–**H**) passing through the same lymphoid follicle from a paraffin-embedded rabbit PP showing an L-IFEB (white arrows). The plane of sectioning, perpendicular to the mucosal surface, moves toward the periphery of the lymphoid follicle from (**A**) to (**H**). The spatial development of the L-IFEB can be easily followed through the sections, from a small bud of cells in A to a crypt-like tubule located between two crypts of the FAE (c1 and c2) in (**G**,**H**). Some cells with an intracytoplasmic lumen (arrowheads) can be appreciated in (**A**,**C**). Cordons of cells outline apparently closed compartments (example in (**E**)). However, adjacent sections demonstrate that the same compartments indeed frequently open into the dome area (black arrow in (**D**)). Crescent-like-shaped cordons of cells with intraepithelial infiltrating immune cells are also visible (black arrow in (**F**,**G**)). A fragment of the regular FAE (X) as seen in (**E**) could be mistakenly identified as an intrafollicular arrangement. However, consecutive sections in (**C**,**D**) reveal it as a mere plain artifact by its continuity with the FAE. Magnification bars = 60 μm. (**I**,**J**) Three-dimensional models of the IFEB (in green), as rendered by 3Dmod software (version 5.1.0). IFEB is seen from two different perspectives. In (**J**), the regular FAE has been also modeled (in blue) to show its spatial relationships with the IFEB.

The planes of sectioning of the PP tissue were either parallel or perpendicular to the mucosal surface. The canonical dome shape, as showed in many review papers, was observed only when the follicle was cut along perpendicular planes passing through its apex. As the cutting planes moved toward the periphery of the follicle, the dome area and the FAE showed an increasingly more irregular shape, characterized by epithelial foldings with a villus-like appearance (Figure 1). This was the result of the presence of furrows running along the sides of the dome. On perpendicular sections, the irregular shape of the PPs led to the identification of portions of the FAE embedded within the lymphoid tissue and apparently isolated from the FAE (Figure 1E). However, serial sectioning of the area clearly showed that these portions of epithelium were indeed in continuity with the FAE, and their isolated appearance was the result of mere plane artifacts (Figure 1C–E). These segments of FAE maintained their regular aspect, characterized by columnar epithelial cells surrounding a narrow lumen in continuity with the intestinal one. On the other hand, we also observed epithelial arrangements with diverse features.

The latter structures formed labyrinth- or micropseudocyst-like structures, or they could be found also as isolated cells in the dome area. The frequency of these IFEBs was as follows: nine out of eleven examined PPs (81.8%) showed at least one of these arrangements. On average, for each PP, we consecutively sectioned 15.86 ± 9.97 (mean ± SD) lymphoid follicles. Overall, pooling together results from all animals, we found IFEBs in 32.81 ± 22.26% (mean ± SD) of follicles. All animals did show the presence of IFEBs. The greater part of the IFEBs were micropseudocyst-like (76.77% M-IFEBs vs. 23.23% L-IFEBs). Apparently, L-IFEBs and M-IFEBs were mutually exclusive, as in our series, we did not observe both types of structures within the same follicle. We noted that only 2 PPs out of 11 seemingly lacked IFEBs. Indeed, analysis of consecutive sections from at least 30 lymphoid follicles for each PP did not show any IFEBs.

### 2.1. Labyrinth-like IFEBs

**Figure 2:**
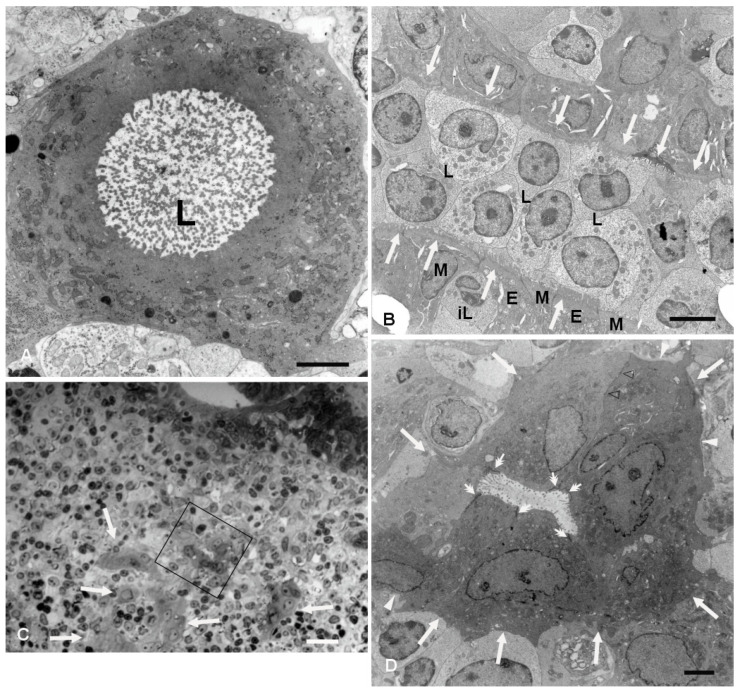
L-IFEBs. (**A**) TEM micrograph of an epithelial cell belonging to a L-IFEB provided with a large intracytoplasmic lumen (L). Magnification bar = 2 μm. (**B**) TEM micrograph showing two cordons of epithelial cells (white arrows pointing to their apical domain) outlining a lumen filled with lymphocytes (L). The lower cordon of cells displays both enterocyte-like cells (E) with long and packed microvilli and M-cell-like cells (M) with shorter cytoplasmic extensions resembling M-cell microfolds. One M-cell-like cell is in contact with two infiltrating lymphocytes (iL) in the proximity of the apical domain of the epithelium. Magnification bar = 5 μm. (**C**) Semithin section intercepting a L-IFEB. Cells of the arrangement differ from immune cells of the dome area for their epithelioid aspect (arrows). The framed area can be seen on a consecutive section at higher magnification in (**D**). Magnification bar = 20 μm. (**D**) TEM micrograph of the framed area in (**C**) taken from a consecutive ultra-thin section confirms the epithelial nature of the IFEBs. Epithelial cells, surrounding a closed lumen with a few scattered microvilli projecting into it, are joined by tight junctions (double white arrows) and desmosomes (empty arrowheads). The basal domain of the cells is mostly without BM, allowing immune cells of the dome area to be in direct contact with the plasma membranes of epithelial cells (white arrows). Small fragments of BM, however, can still be seen (white arrowheads). Magnification bar = 3 μm.

Labyrinth-like IFEBs (L-IFEBs) were characterized by complex arrangements within the dome area of the lymphoid follicles. These arrangements seemed to arise as small buds of epithelial cells from the bottom of the furrows running along the dome (Figure 1A). Moving the cutting plane toward the periphery of the dome, serial sections of the tissue showed the buds developing into complex and convoluted structures mostly detached from the FAE (Figure 1B–E). L-IFEBs were formed by branched cordons of cells outlining irregular lumina with a labyrinth-like arrangement (Figure 1C). In some cases, the closed lumen of L-IFEBs appeared completely filled with lymphoid cells (Figure 1E). Actually, consecutive sections of the same areas showed that the lumen frequently opened into the dome area of the lymphoid follicle (Figure 1D). In such arrangements, some cordons of cells outlined crescent-shaped fragments of epithelium opened towards the dome area (Figure 1F,G). Also, epithelial cells forming the L-IFEBs ranged from typical columnar cells (Figure 1E) to flat cells (Figure 1D). In some cases, cells appeared to have lost their regular polarity with the formation of intracytoplasmic lumina (Figure 1A,C). The latter feature could be better appreciated by transmission electron microscopy (TEM), which demonstrated cells containing a single large vacuole with microvilli protruding into it (Figure 2). As the cutting plane moved toward the most peripheral area of the dome, L-IFEBs decreased in size, eventually resolving into crypt-like structures (Figure 1G,H). By TEM, it was possible to note that a continuous basement membrane (BM) was absent along large portions of the structures, allowing direct epithelial–lymphoid cells interactions (Figure 2B,D).

Cells forming L-IFEBs showed a diverse morphology compared to the regular FAE, with some of them displaying features of undifferentiated cells (large nuclei with fine and dispersed chromatin, scattered and variable-sized microvilli). Nevertheless, the epithelial nature of these cells was always evident, as they displayed well-developed intercellular junctions and microvilli that projected into the lumen when the latter was present (Figure 2B). In some areas of the L-IFEBs, M-cell-like cells could be observed, with short and scattered microvilli resembling M-cell microfolds. These microfold-like apical cytoplasmic protrusions were clearly distinct from the longer and more packed microvilli of the adjacent enterocytes (Figure 2B). Even though we did not have the chance to observe large pockets filled with immune cells under the apical membrane of these cells, lymphocytes could be seen finding their way between epithelial cells, always in contact with the M-cell-like cell membrane (Figure 2B).

**Figure 3:**
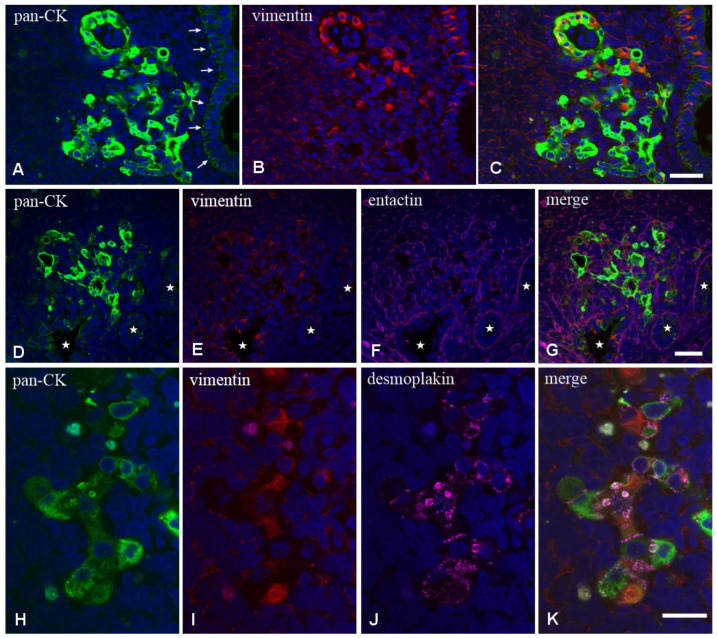
Laser scanning confocal microscopy of L-IFEBs. (**A**–**C**) Double labeling with the antibodies anti-panCK (green) and anti-VIM (red). Note the great difference of CK-immunoreactivity between the regular FAE (arrows) and the L-IFEB. The signal intensity of L-IFEB in the green channel is so strong that it saturates the confocal detector. Some cells of the L-IFEB are CK/VIM double-immunoreactive cells. (**D**–**H**) Triple labeling with the antibodies anti-panCK (green), anti-VIM (red) and anti-entactin (magenta). Compared to (**A**–**C**), confocal microscope settings have been adjusted to avoid signal saturation from the L-IFEB. However, the epithelium of two nearby crypt-like structures and one portion of the regular FAE (white stars) becomes barely visible. Whereas the BM component entactin regularly underlies the FAE, under the cells of the L-IFEB, it appears discontinuous. Magnification bars = 50 μm. (**H**–**K**) Triple labeling with the antibodies anti-panCK (green), anti-VIM (red) and anti-desmoplakin (magenta). Cordons of cells, made by panCK^+^ intestinal epithelial cells and panCK^+^/VIM^+^ cells, are joined together by several desmosomes, as unveiled by desmoplakin-immunoreactive spots on the cell surface. Magnification bar = 20 μm.

We then analyzed frozen sections by confocal microscopy to ascertain the expression of epithelium-specific markers, such as cytokeratin (CK) and junction-associated proteins desmoplakins and zonulin-1 (ZO-1), or BM markers such as entactin. Experiments with the antibody anti-panCK showed that the L-IFEBs contained CK-immunoreactive cells. Interestingly, CK-immunoreactivity in L-IFEBs appeared much stronger compared to the regular FAE, as if CK intermediate filaments underwent a compacting process, or they were simply more represented (Figure 3A–C). Most importantly, we also assessed the presence of vimentin (VIM)/panCK double-immunoreactive cells, a feature of rabbit M cells in the regular FAE [6]. Staining with anti-entactin antibodies, on the other hand, showed only a discontinuous BM, corroborating previous observations by TEM (Figure 2). Indeed, entactin appeared absent in large portions of L-IFEBs (Figure 3D–G). Finally, desmoplakins-immunoreactive dot-like labeling on the surface of CK-immunoreactive cells demonstrated the presence of desmosomes (Figure 3H–K).

### 2.2. Micropseudocyst-like IFEB

**Figure 4:**
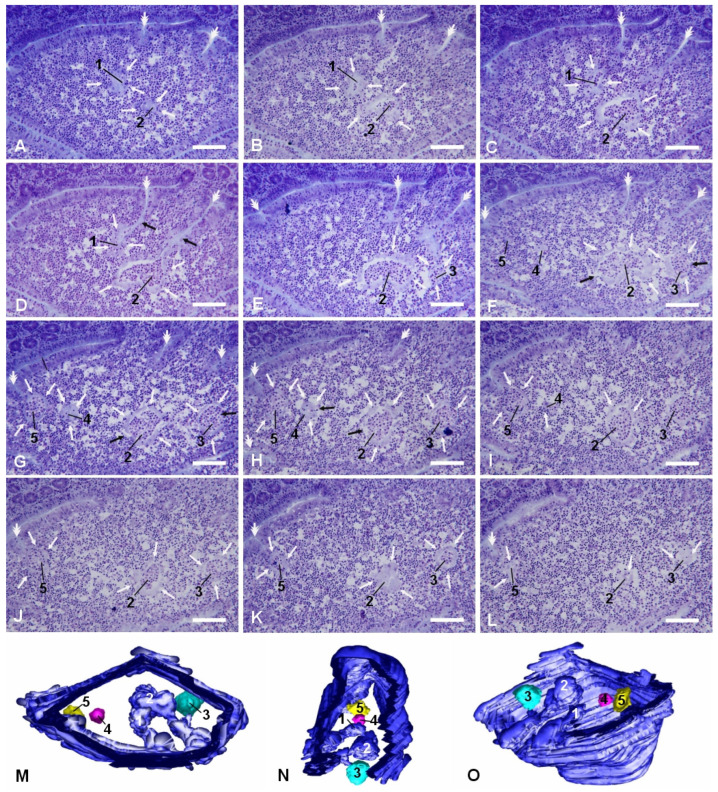
M-IFEBs. Selected slides from a series of consecutive sections (**A**–**L**) passing through the same lymphoid follicle from a methylmethacrylate-embedded rabbit PP demonstrating 5 M-IFEBs. The level of sectioning moves parallel to the surface of the mucosa from the more superficial (**A**) to deeper planes (**L**). The M-IFEBs (white arrows) have been numbered from 1 to 5. The first two arrangements (1 and 2) are seen from A to C as IFEBs filled with immune cells. However, they are still connected with the intestinal lumen through narrow tubes (black arrows in (**D**)) that open at the bottom of the superficial furrows (white double arrows) engraving the surface of the dome. Whereas M-IFEB number 1 soon disappears (it is already absent in (**E**)), M-IFEB number 2 is visible throughout the entire series of sections. The other three M-IFEBs appear in sections (**E**) (number 3) and (**F**) (numbers 4 and 5), and they do not show any connection with the intestinal lumen. M-IFEBs numbers 3 and 4 maintain their separation from the FAE throughout their entire sectioning, whereas M-IFEB number 5 is still in contact with the bottom of a furrow, though its lumen seems sealed. M-IFEBs numbers 2 to 4 show interruptions in their wall (black arrows in (**F**–**H**)), and their lumen is in continuity with the dome area. Magnification bars = 25 μm. (**M**–**O**) Three-dimensional rendering of the same M-IFEBs (3Dmod software, version 5.1.0), as seen from different perspectives. Two of them (numbers 2 and 4) are still in continuity with the FAE and, accordingly, have the same color (blue) of the FAE; (**M**) The dome is seen from above, and the apical FAE has been clipped away; (**N**) The dome is seen from one side, and the lateral FAE has been clipped away; (**O**) The dome, seen from below, has been slightly tilted to facilitate M-IFEBs identification.

More frequently, we observed smaller and more regularly shaped epithelial arrangements in the dome areas of PP lymphoid follicles. They appeared as small rounded arrangements of flattened epithelial cells that could be either in direct relation with the FAE, with their lumen in continuity with the intestinal one, or completely detached from it (Figure 4). These micropseudocystic-like IFEBs (M-IFEBs) were usually filled with lymphoid cells (Figure 4) or with luminal debris. Sometimes, they showed disruptions of the continuity in the epithelial lining, leading to direct connections between their lumen and the surrounding lymphoid tissue (Figure 4F–H). M-IFEBs resembled a sort of epithelial bubbles that seemed to bud from the bottom of the farrows running along the dome (Figure 4G).

**Figure 5:**
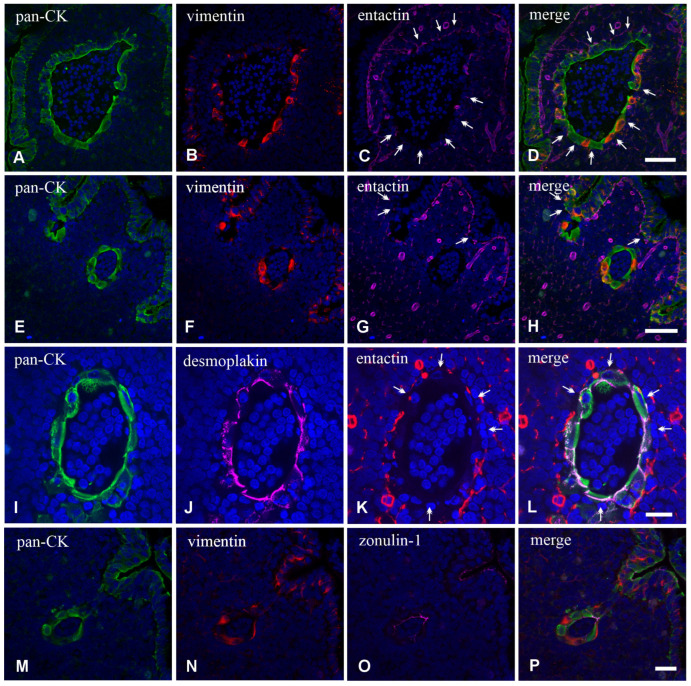
Laser scanning confocal microscopy of M-IFEBs. (**A**–**H**) Triple labeling with the antibodies anti-panCK (green), anti-VIM (red) and anti-entactin (magenta). Two M-IFEBs are filled with cells and are lined by a simple flattened epithelium made by CK+ epithelial cells and CK+/VIM+ M-cell-like cells. The M-IFEB BM in (**A**–**D**) is stained with the anti-entactin antibody and appears absent in the lower part of the arrangement (white double arrows), whereas it is present, though discontinuous, on the upper part (white arrows). Note the regular and continuous basement membrane under the FAE. BMs are stained with the anti-entactin antibody also in (**E**–**H**), and, even in this case, the M-IFEB appears unlabeled. In contrast to (**A**–**D**), however, note that the regular BM under the FAE is interrupted (double white arrows) under the epithelium lining the furrows that engrave the dome. Magnification bars = 60 μm. (**I**–**P**) Intercellular junctions in M-IFEBs. Triple-labeling experiment with the antibodies anti-panCK (green), anti-entactin (red) and anti-desmoplakin (magenta) in (**I**–**L**) shows that cells are joined together by several desmoplakin^+^ desmosomes. BM is discontinuous also in this sample. Magnification bar = 30 μm. Triple-labeling experiment with the antibodies anti-panCK (green), anti-VIM (red) and anti-ZO-1 (magenta) in (**M**–**P**) demonstrates that cells are still provided with anti-ZO-1^+^ tight junctions. Magnification bar = 20 μm.

Similarly to L-IFEBs, the M-IFEBs displayed one layer of CK-immunoreactive cells, with some interspersed CK^+^/VIM^+^ M-cell-like cells (Figure 5A–H,M–P). Unlike L-IFEBs, however, CK-immunoreactivity in M-IFEBs epithelial cells was comparable to that observed in the regular FAE (Figure 5A–H). We also observed that an entactin-immunoreactive BM was partially (Figure 5A–D,I–L) or totally (Figure 5E–H) absent along the IFEBs circumference. Further, cells of these structures were joined by dot-like desmoplakins-immunoreactive spots (Figure 5I–L) and by ZO-1-immunoreactive belts (Figure 5M–P), demonstrating the presence of desmosomes and tight junctions. In most cases, the lumina of the M-IFEBs were filled with CK^−^/VIM^−^ cells (Figure 5).

### 2.3. Scattered Isolated Epithelial Cells in the Dome Area and Absence of Cellular Apoptosis

**Figure 6:**
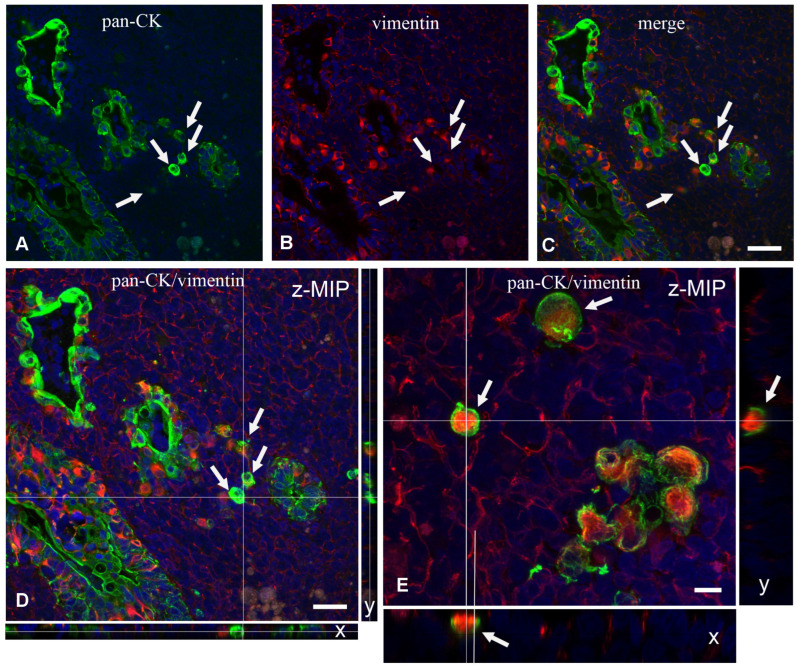
Laser scanning confocal microscopy of scattered intrafollicular isolated epithelial cells. (**A**–**C**) Double-labeling experiment with the antibodies anti-panCK (green) and anti-VIM (red) shows a couple of M-IFEBs associated with a few apparently isolated nearby scattered cells (white arrows). To demonstrate that such cells were really isolated, we acquired a Z-stack series of images through the entire thickness of the section. (**D**) Z-projection of the entire stack (Z maximal intensity projection = z-MIP) and y and x projections of the planes passing through the white lines drawn in z-MIP. White lines in y and x refer to the level of the focal plane shown in (**A**–**C**). y and x projections demonstrate that the intercepted cells (white arrows) are indeed isolated and not in contact with other epithelial cells. Magnification bars 30 μm. (**E**) Double-labeling experiment with the antibodies anti-panCK (green) and anti-VIM (red) shows a fragment of an M-IFEB associated with a couple of apparently isolated nearby scattered cells (white arrows). Z-stack series of images through the entire thickness of the section, which is shown as z-MIP. White lines in z-MIP refer to x and y planes that intercept one isolated cell and that are shown beside and below z-MIP. y and x projections demonstrate that the intercepted cell is indeed isolated and not in contact with other epithelial cells. Magnification bar 10 μm.

In the vicinity of the IFEBs, it was often possible to observe individual CK^+^ or CK^+^/VIM^+^ double-labeled cells within the dome area without any apparent contiguity with other epithelial cells (Figure 6A–C). This was confirmed by analysis of long Z-stack images by confocal microscopy. Although at times we observed that the apparently isolated cell was indeed part of small tubular branches of L-IFEBs, analysis of Y and X projections of Z-stacks confirmed that some of them were indeed isolated (Figure 6D,E). Finally, we evaluated whether IFEBs underwent cellular apoptosis. Terminal dUTP Nick End Labeling (TUNEL) analysis did not show any apoptosis either in the above-mentioned structures or in the isolated cells that we could evaluate.

## 3. Discussion

Here, we describe the existence of IFEBs hosted in the dome area of rabbit PP lymphoid follicles, and that, to our knowledge, has never been previously reported. Two types of IFEBs were observed: M- and L-IFEBs. In addition to these structures, isolated CK^+^ epithelial cells were also found scattered in the dome area and close to the IFEBs. The observations that we have gathered suggest that the M-IFEBs are generated from the FAE likely following a multistep course. This, eventually, leads to a complete disassembling of the structures, resulting in the presence of isolated epithelial cells within the lymphoid tissue of the PP. First, a short portion of the FAE invaginates at the bottom of the furrows that run along the side of the dome and forms short tubules with their lumens still in continuity with the intestinal one. Possibly, the very first step in the generation of M-IFEBs is the degradation of the epithelial BM. This is suggested by the observation that furrows of the FAE adjacent to micropseudocyst-like structures did not express an entactin-immunoreactive BM. The second step is the sealing of the opening of the FAE invagination, resulting in the formation of an epithelial bubble-like isolated structure that can be either empty or contain luminal debris. The L-IFEB then moves away from FAE and progresses within the lymphoid tissue as an isolated structure. As it detaches from the FAE and relocates into the dome area, the FAE-derived structure is infiltrated by cells of the local microenvironment that mechanically enlarge the lumen, distending epithelial cells and leading to the rupture and disassembling of the IFEB. Eventually, this will end with the presence of intrafollicular isolated individual epithelial cells. The mechanism through which lymphoid cells fill the lumen of these structures is not clear, even though the presence of M-cell-like cells could suggest a transcytotic passage, as it regularly occurs when immune cells migrate from the dome into the intestinal lumen [16]. Although the breaking of the micropseudocysts is likely a late event, it is also possible that infiltration may occur directly through the openings that develop between epithelial cells.

The formation of L-IFEBs is more puzzling. One possibility is that they might arise from the FAE with a mechanism similar to the one proposed for M-IPEBs but in areas located much closer to the FAE crypt. Alternatively, the entire structure could develop directly from an associated crypt. In this case, we should envision a more stable structure, in continuity with the intestinal lumen through very narrow openings in the crypt wall.

The average incidence of the IFEBs within PPs is relatively high, although their frequency appears extremely variable, reaching 33 ± 22.26% (mean ± SD) in all follicles examined (n = 11). This suggests that they are likely short-lived structures that may serve only in particular functional moments of the lymphoid follicles. It will be interesting to verify whether their formation is triggered by antigenic challenge, as for other morphological changes occurring in the FAE [8,10,11]; dietary factors; or they just develop spontaneously as an intrinsic activity of the FAE. However, given the age of the animals examined, at this time, we cannot rule out the possibility that these structures are restricted to young animals.

A particularly intriguing observation is that most of the IFEBs including isolated epithelial cells lost large portions of their BMs without displaying evident ultrastructural signs of apoptosis. Indeed, besides not observing any apoptotic body, we could not detect apoptotic cells by the TUNEL test. Anchorage-related apoptosis (anoikis) is a well-documented and long known phenomenon involving epithelial and endothelial cells [17,18,19]. Thus, it is quite surprising to find non-tumoral epithelial cells without BM not undergoing apoptosis. In other words, cells of the IFEBs, at least those of the M-IFEBs, become as resistant to anoikis as tumor cells are. Integrins, receptors of the various components of the extracellular matrix, are heavily involved in anoikis, which together with the cytoskeleton, seems to drive survival signals from the outside inside the cells [20]. However, there are examples of cells that are rescued from anoikis via different mechanisms. Epithelial cells can be rescued from apoptosis by preserving the integrity of cell–cell adhesions [21], as it occurs in cells of the M-IFEBs and L-IFEBs. Another possibility for receiving survival signals is represented by the particular microenvironment in which these epithelial structures are located, where they can be exposed to a large set of cytokines and growth factors. Indeed, growth factors such as insulin-like growth factors (IGFs), transforming growth factor (TGF)-β1 and macrophage stimulating protein can activate the phosphoinositide 3-kinase/RAC-alpha serine/threonine-protein kinase (PI-3K/Akt-1) pathway, which is known to be instrumental for cell survival and anoikis resistance [22,23,24,25]. Tumor cells can also avoid anoikis acquiring a rounded shape and forming blebs, which are important to induce resistance to anoikis [26].

However, the ability to escape apoptosis by cells of the IF structure could be discussed by examining the similarity with recently described intracellular organelles that might confer resistance to apoptosis. Some cells have been reported to form unilocular giant vacuoles as key structures capable of inducing anoikis-resistance [27]. This organelle carries similarity to the intracytoplasmic lumen that we observed in some cells of the IFEBs. Interestingly, the formation of giant vacuoles requires F-actin depolymerization and the involvement of proteins of the septin family, the same family of proteins required for bleb formation and anoikis resistance in tumor cells [26]. On the other hand, intracytoplasmic lumen in itself may be relevant to inducing resistance to anoikis, as it is a feature associated with apoptosis-resistant tumor cells [28,29,30,31,32].

Interestingly, a recently described organelle termed apicosome has been implicated in establishing cell polarity in human pluripotent stem cells [33]. Like giant unilocular vacuoles, apicosomes bear a strong resemblance to the intracytoplasmic lumen observed in IFEBs. Thus, giant unilocular vacuoles, intracytoplasmic lumina and apicosomes could be part of a strategy to preserve and confine the apical domain of the cell membrane when perturbations of cell polarity occur or when cell polarity is about to be established. At any rate, the intracytoplasmic lumen of IFEB cells fits well in the context of cells whose polarity is perturbed by missing contact with the BM. The main limitation of this study is that the function of IFEBs still remains to be determined, although their presence in all animals used points to these structures being of certain biological relevance. Also, although this was beyond the aim of our investigation, we are not in the position to report whether these newly described structures could be found or not in older animals. The latter notion could be instrumental for the formulation of hypotheses on their immunological function late in life. Indeed, it is not even certain that M-IFEBs and L-IFEBs share the same functions. Whereas M-IFEBs are likely transient and dynamic structures as they lose continuity with the FAE, L-IFEBs may be more stable elements. Possibly, the role of these structures could be envisioned by analyzing the phenotype of the immune cells that are in contact or inside their lumina. Unfortunately, studies along this line are hampered by the lack of available antibodies against rabbit immune markers. In this respect, the finding of IFEBs in mice or humans could help to shed lights on their functions.

## 4. Materials and Methods

### 4.1. Antibodies and Reagents

Mouse anti-vimentin mAb (clone V9, code V6389) and a cocktail of anti-pancytokeratin mAbs (a mixture of c-11, PCK-26, CY-90, KS-1A3, M20, A53-B/A2 clones, code C2562) were purchased from Sigma-Aldrich (Milan, Italy). A cocktail of biotinylated mouse monoclonal antibodies anti-pancytokeratin (code NBP2-76425B) was from Novus Biologicals (Cambridge, UK). Goat anti-vimentin antibody and mouse anti-Zonulin-1 mAb (clone T8-754) were kind gifts, respectively from Peter Traub (Max Planck Institut für Zellbiologie, Rosenhof, Ladenburg, Germany) [34] and from Mikio Furuse (Department of Cell Biology, Kyoto University, Kyoto, Japan) [35]. A multi-epitope cocktail of mouse anti-desmoplakin 1&2 mAbs (clones DP1&2-2.15, DP1-2.17, DP1&2-2.20, code RDI-PRO65146) was purchased from Research Diagnostics Inc. (Flanders, NJ, USA). Goat anti-nidogen-1/entactin antibody (code AF-2570) was from R&D systems (Minneapolis, MN, USA).

Positive immunoreactions with the above-mentioned antibodies were unveiled with the following secondary reagents: donkey anti-goat rhodamine-conjugated IgGs (code AP180R) from Chemicon (Temecula, CA, USA), donkey Cy5-conjugated F(ab’)2 fragment anti-mouse IgGs (code 715-176-150), donkey FITC-conjugated F(ab’)2 fragment anti-mouse IgGs (code 715-096-151) and Dylight 488-conjugated streptavidin (code 016-480-084) from Jackson ImmunoResearch laboratories Inc. (West Grove, PA, USA).

The Roche In Situ Cell Death Detection Kit was purchased from Sigma-Aldrich (Milan, Italy).

### 4.2. Animals and Tissues

Five young New Zealand rabbits (age range 8 to 20 weeks old) were used for the collection of the PP tissues, according to the Guiding Principles in the Use of Animals and accepted by the Animal Ethics Committee of the University of Siena (authorization #265/2018-PR, ISOPRO 7DF19.23 (to G.C.)). Two rabbits were 8 weeks old, two rabbits were 12 weeks old and one rabbit was 20 weeks old. After sacrifice, PPs were promptly frozen in liquid-nitrogen pre-chilled isopentane, as previously reported [36,37], and stored at −80° until use. Fragments of PPs, fixed in 0.8% paraformaldehyde and 2% glutaraldehyde in 0.2 M cacodylate buffer (pH 7.35) for 3 h at 4° and post-fixed in 1% OsO4 in 0.1 M cacodylate buffer for 2 h at 4°, were processed for TEM and embedded in Epon-812.

Also, some tissues were processed according to standard procedures, and PPs were fixed by immersion in Bouin’s fluid for 12–24 h at room temperature or in 2% glutaraldehyde diluted in 0.2 M cacodylate buffer (pH 7.35) for 6 h at 4 °C. Bouin-fixed PPs were treated for conventional paraffin embedding procedures, whereas glutaraldehyde-fixed PPs were processed for routine embedding in methylmethacrylate (Technovit 8100 Kulzer; Wehrheim, Germany).

### 4.3. Light and Transmission Electron Microscopy

Light microscopic studies were carried out on long series of consecutive sections obtained from both paraffin- and methylmethacrylate-embedded tissues cut along two orthogonal planes: parallel to the mucosal surface, from the tip of the villi to the germinal centers of the lymphoid follicles, or perpendicular to the mucosal surface. Sections (3 μm thick) of PPs from methylmethacrylate blocks were stained with 0.1% toluidine blue; sections (4–5 μm thick) from paraffin blocks were stained with hematoxylin and eosin. Sections (20 μm thick) from frozen samples were fixed for 10 min in acetone at −20° and used for immunofluorescence experiments. Series of sections were viewed with 3Dmod software (version 5.1.0, University of Colorado, Boulder, CO, USA). Models of IFEBs were created by manually drawing epithelial contours on consecutive sections. Surface models were obtained with the same software.

Semi-thin sections (0.5 μm thick) from epon-embedded samples were stained with 0.1% toluidine blue. Areas of interest were spotted, and consecutive ultra-thin sections were carried out and mounted on cupper grids. After staining with uranyl acetate and lead citrate, grids were examined using a Philips 201 electron microscope (Philips, Eindhoven, The Netherland).

### 4.4. Immunofluorescence

The following set of multiple labeling experiments was carried out: (1) mouse anti-vimentin mAb followed by donkey Cy5-conjugated anti-mouse IgGs, mouse biotinylated anti-panCK mAb followed by Dylight 488-conjugated streptavidin, goat anti-entactin antibody followed by donkey anti-goat rhodamine-conjugated IgGs; (2) mouse anti-desmoplakin mAb followed by donkey Cy5-conjugated anti-mouse IgGs, mouse biotinylated anti-panCK mAb followed by Dylight 488-conjugated streptavidin, goat anti-entactin antibody followed by donkey anti-goat rhodamine-conjugated IgGs; (3) mouse anti-ZO-1 mAb followed by donkey Cy5-conjugated anti-mouse IgGs, mouse biotinylated anti-panCK mAb followed by Dylight 488-conjugated streptavidin, goat anti-vimentin antibody followed by donkey anti-goat rhodamine-conjugated IgGs; (4) goat anti-vimentin antibody followed by donkey anti-goat rhodamine-conjugated IgGs, mouse anti-panCK mAb followed by donkey FITC-conjugated anti-mouse IgGs. Before the application of each antibody, sections were blocked with 5% bovine serum albumin in phosphate-buffered saline for 10 min. Cell nuclei were always counterstained with 1 μM 4′,6-diamidin-2-phenylindole (DAPI) for 8 min at the end of all experiments. Negative controls were carried out with the same procedures but omitting the primary antibodies. Relevant images were acquired using an FV3000 laser scanning confocal microscope (Evident, Hamburg, Germany).

### 4.5. TUNEL Test

In order to identify apoptotic cells, a TUNEL assay was used on frozen sections. Briefly, sections were fixed in 4% paraformaldehyde in 0.1 M phosphate buffer (pH 7.4) for 20 min. Then, the TUNEL reaction was carried out according to the manufacturer’s instructions. Positive controls were obtained by previously incubating slides for 10 min with 300 U/mL DNase I.

## Data Availability

Data is contained within the article

## Abbreviations

The following abbreviations are used in this manuscript:

PP	Peyer’s patches
BM	Basement membrane
CK	Cytokeratin
FAE	Follicle-associated epithelium
IFEB	Intrafollicular epithelial body
IGF	Insulin-like growth factor
L-IFEB	Labyrinth-like intrafollicular epithelial body
M-cell	Membranous cell
M-IFEB	Micropseudocystic-like intrafollicular body
PI-3K/Akt-1	Phosphoinositide 3-kinase/RAC-alpha serine/threonine-protein kinase
RANKL	Receptor activator of nuclear factor κ-Β ligand
TEM	Transmission electron microscopy
TGF-β1	Transforming growth factor-β1
TUNEL	Terminal deoxynucleotidyl transferase (dUTP) nick end labeling
VIM	Vimentin
ZO-1	Zonulin-1
